# Microecological modulators—new perspectives for treating sex hormone disorders

**DOI:** 10.3389/fmicb.2026.1795152

**Published:** 2026-04-30

**Authors:** Xinhua Li, Xiaoyan Yu, Yihan Zhong, Guihua Zhou, Yinggang Zou

**Affiliations:** 1 The Second Hospital of Jilin University, Jilin University, Changchun, China; 2 School of Pharmaceutical Sciences, Jilin University, Changchun, China; 3 Heersink School of Medicine, University of Alabama at Birmingham, Birmingham, AL, United States

**Keywords:** endometriosis, gut microbiota-sex hormone axis, polycystic ovary syndrome, probiotics, prostate cancer, sex hormone-related tumors

## Abstract

Sex hormone related disorders, characterized by complex etiology and long-term health risks, pose a significant challenge to global health. Hormone-based therapies are often accompanied by adverse effects and fail to address the underlying pathophysiological mechanisms. The “gut microbiota-sex hormone axis” maintains endocrine homeostasis through diverse pathways, including enzymatic reactions, immune modulation, metabolic regulation, and the microbiome-gut-brain axis. Dysregulation of this axis has been identified as a critical factor in the pathogenesis of sex hormone-related disorders. Probiotics have emerged as a promising adjunctive therapeutic strategy by targeting this axis. Preclinical and clinical studies have demonstrated that specific probiotic strains ameliorate hormonal imbalances, attenuate inflammation, and optimize metabolic parameters, showing positive efficacy in sex hormone-related disorders. This review systematically elaborates the regulatory mechanisms of this bidirectional axis and highlights the application of probiotics and its regulatory roles as targeted interventions in related disorders.

## Graphical Abstract

**Figure:**
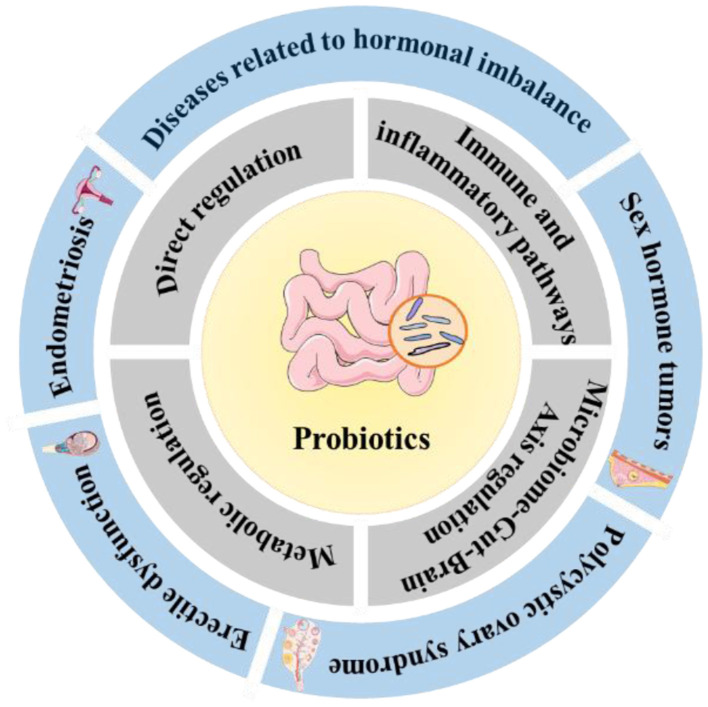

## 1 Introduction

The gut microbiota comprises over 100 trillion microorganisms. It is the largest microbial system with a biotransformation capacity which is comparable to the liver (Guo et al., 2016). It is involved in host physiological or pathological signaling pathway, and also play a key role in disease development. Therefore, it has been defined as a virtual organ (Turnbaugh et al., 2007). In recent years, the complex interplay between the gut microbiota and sex hormones has attracted attention in multiple fields such as endocrinology, microbiology, and immunology (Haag et al., 2012).

Studies have revealed that sex hormones can influence the intestinal physical barrier, immune milieu, and microbial composition, while the gut microbiota, in turn, modulates sex hormone levels (Wu et al., 2024c). A novel concept of “gut microbiota-sex hormone axis” has been defined (Flak et al., 2013), delineating the bidirectional interactions between sex hormones and the gut microbiome (Kim et al., 2020; Wu et al., 2024b).

On one hand, the gut microbiota can regulate sex hormone levels through direct involvement in the synthesis of hormone-related enzymes and/or indirect modulation of inflammatory, immunity, and metabolic pathways (Antwis et al., 2019). For instance, gut microbes secrete β-glucuronidase (GUS), which regulates estrogen production and facilitates the reabsorption of free sex hormones during enterohepatic circulation, thereby maintaining systemic hormone levels and their downstream physiological effects (Wu et al., 2024c). Additionally, glucocorticoids can be converted into androgens by specific gut bacteria, such as *Clostridium scindens* (Lv et al., 2024). On the other hand, sex hormones can modulate bile acid metabolism, immune status, and gut barrier function, leading to distinct sexual dimorphism in gut microbial structure that evolves dynamically across life stages (Chen et al., 2021). For example, specific deletion of estrogen receptor β (ERβ) alters the gut microbiota composition in mice (Ibrahim et al., 2019). Research has revealed that chronic inflammation, metabolic disturbances, or immune homeostasis due to genetic, environmental, dietary, or pharmaceutical factors could result in dysregulation of the “gut microbiota-sex hormone axis,” which may promote the pathogenesis of related disorders (Liang et al., 2024; Raimondi et al., 2025). Those disorders include female reproductive endocrine diseases (Salehi et al., 2024; Tang et al., 2024; Qiao et al., 2024; Deng et al., 2024; Zhu et al., 2025; Eslami et al., 2025; Xing et al., 2024), and other sex-hormone-related conditions (Rosa et al., 2020).

Probiotics have garnered increasing attention as a microecological intervention strategy due to their multi-targeted effects and favorable safety profile (Barba et al., 2020). Their mechanisms of action are multifaceted, encompassing intestinal adhesion, competitive exclusion of pathogens for nutrients and receptor binding sites, reinforcement of mucosal barrier integrity, immunomodulation, and the production of bacteriocins and signaling molecules (Sassone-Corsi and Raffatellu, 2015). Probiotics primarily exert their effects through regulation of the gut microbiota–sex hormone axis to balance sex hormone levels. This includes modulating microbial community structure to influence enterohepatic circulation (Wang et al., 2020), directly metabolizing or transforming sex hormones (Zhang et al., 2019). Probiotics also alleviate inflammation and oxidative stress (Mehdizadeh et al., 2022), and regulate the hypothalamic-pituitary-gonadal axis via the microbiome-gut-brain axis (MGBA) (Haas et al., 2020). By restoring intestinal microecological homeostasis, probiotics can indirectly or directly correct dysregulation of the gut microbiota–sex hormone axis, offering novel insights into the prevention and adjunctive treatment of related disorders (Taniya et al., 2022). This review aims to elucidate the mechanistic underpinnings of this bidirectional axis and systematically evaluate the potential and progress of probiotic interventions in modulating associated diseases.

## 2 Mechanisms underlying the gut microbiota-sex hormone axis interactions

### 2.1 Direct interactions between gut microbiota and sex hormone

**Figure 1:**
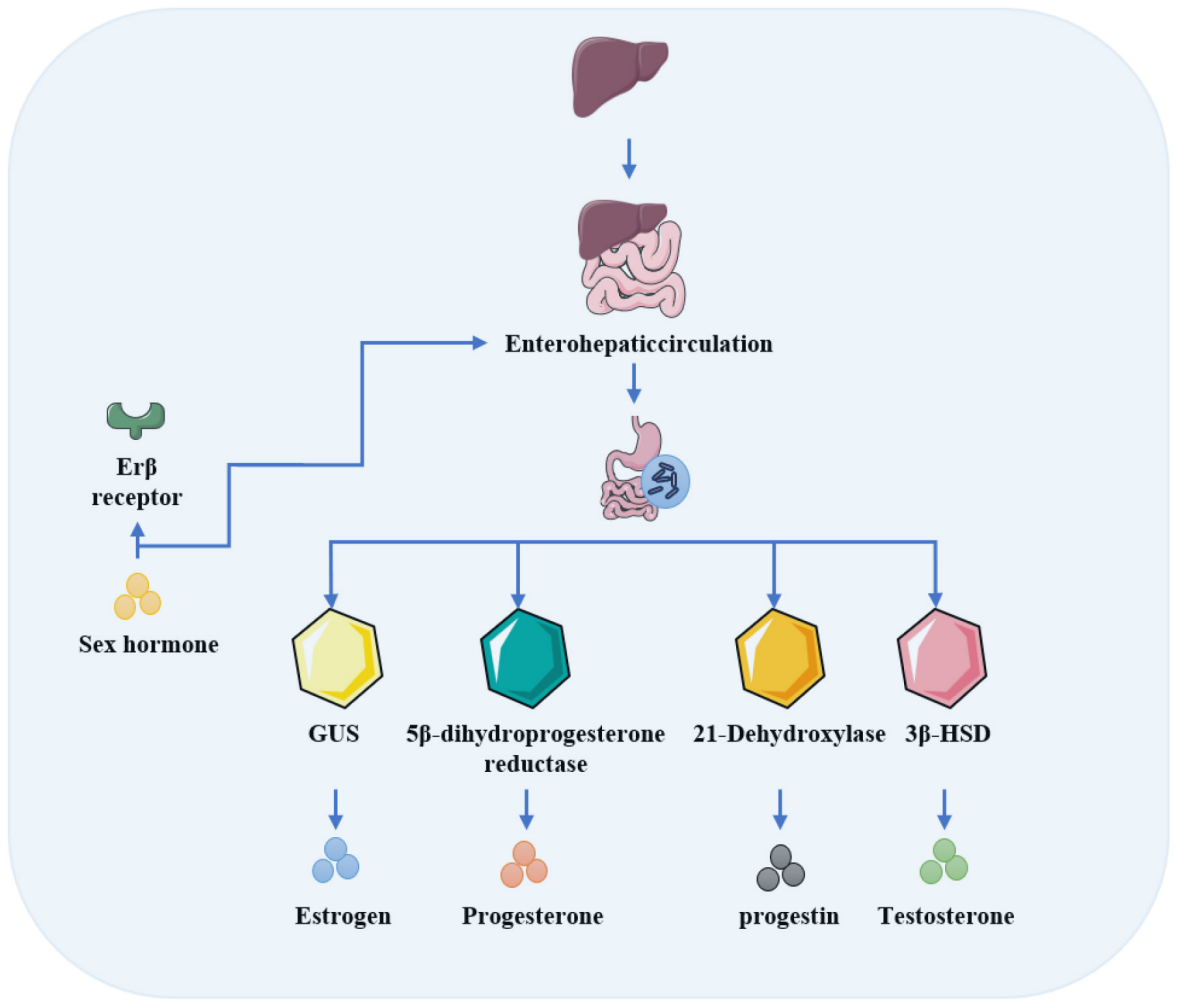
Direct interaction between the gut microbiota and sex hormones.

The interplay between the gut microbiota and sex hormones extends beyond indirect effects mediated by the host immune system or metabolic pathways, encompassing direct molecular mechanisms of interaction. Specifically, the gut microbiota actively participates in sex hormone metabolism through the expression of a diverse array of enzymes, including 3β-hydroxysteroid dehydrogenase (3β-HSD) and GUS. Conversely, sex hormones directly modulate the abundance and composition of the gut microbiota. The schematic diagram illustrating the mechanisms underlying the direct bidirectional interactions between the gut microbiota and sex hormones is presented in Figure 1.

Testosterone is a typical sex hormone regulated by the gut microbiota. Testosterone metabolites are excreted into the intestine via bile. From the intestine, these metabolites can be reabsorbed into the bloodstream, thereby complete the enterohepatic circulation. The gut microbiota directly intervenes in this process by modulating the ratio of active to inactive forms of steroid hormones (Li et al., 2022). In individuals with depression, specific gut microbes expressing 3β-HSD have been identified. These bacteria are capable of directly degrading testosterone therefore leads to reduced serum levels of both testosterone and estradiol. Such findings underscore the critical role of the gut microbiota-sex hormone axis in the pathophysiology of depression (Li et al., 2022, 2023). Furthermore, *Clostridium innocuum*, which harbors NADPH-dependent 5β-dihydroprogesterone reductase, can modulate host progesterone levels by influencing its enterohepatic circulation (Chen M. J. et al., 2024). Estrogen metabolism is similarly regulated by the gut microbiota. Following conjugation with glucuronic acid in the liver, estrogen is secreted into the intestine. Intestinal microbial GUS catalyzes its deconjugation, thereby restoring estrogen bioactivity. Research has revealed dynamic changes in both gut microbiota composition and GUS expression during the human menstrual cycle (Bergstrom et al., 2021).

The gut microbiota can also transform sex hormones in the host body. An incidental finding revealed that hydrogen produced by commensal gut bacteria promotes bacterial 21-dehydroxylation. For instance, *Gordonibacter pamelaeae* and *Eggerthella lenta* convert bile corticosteroids into progestins (Wang Y. et al., 2020). The enhancing effect of hydrogen on this process further accelerates the transformation of steroids into sex hormones and neurosteroids (McCurry et al., 2024). Gut microbes may also directly influence gonadal function. For example, spermidine-enriched *Parabacteroides distasonis* has been shown to ameliorate drug-induced testicular injury and promote spermatogenesis (Zhao et al., 2021). In contrast, Klebsiella strains rich in 3β-HSD can directly degrade estrogen, leading to decreased serum estrogen levels in premenopausal women (Zhao et al., 2021; Li et al., 2023). Certain mucin-degrading bacteria play a crucial role in maintaining the integrity of the mucus barrier (Everard et al., 2013). Once this barrier is compromised, gut bacteria may translocate into the systemic circulation. Such translocation can trigger systemic inflammation, which subsequently inhibits testosterone production by Leydig cells (Tremellen, 2016).

Though it has been well known that the effects of sex hormones on the gut microbiota is mediated indirectly through host immune and metabolic systems, emerging evidence indicates that sex hormones can directly interact with gut microbes, independent of host intermediation (Vriend et al., 2024). Studies have confirmed a significant correlation between sex hormone levels and the diversity and composition of the gut microbiota (Ma et al., 2022). For instance, specific deletion of ERβ in intestinal epithelial cells alters the gut microbiota composition in mice (Ibrahim et al., 2019). Furthermore, a marked decline in estrogen levels enriches gut microbial diversity, resulting in an increase in taxa such as *Bacteroides, Prevotella marshii*, and *Veillonella dispar*, a phenomenon described as a menopausal shift in women's health and microbial niches (Peters et al., 2022). Hyperandrogenism has been shown to alter the gut microbiome in women with polycystic ovary syndrome (PCOS) (Torres et al., 2018). In neonates, masculinized female individuals exhibit distinct gut microbial community changes characterized by higher *Bacteroidetes* and lower *Firmicutes* abundance in early adulthood, though these alterations diminish with age (Barroso et al., 2020).

### 2.2 Immunoregulatory and inflammatory pathways

**Figure 2:**
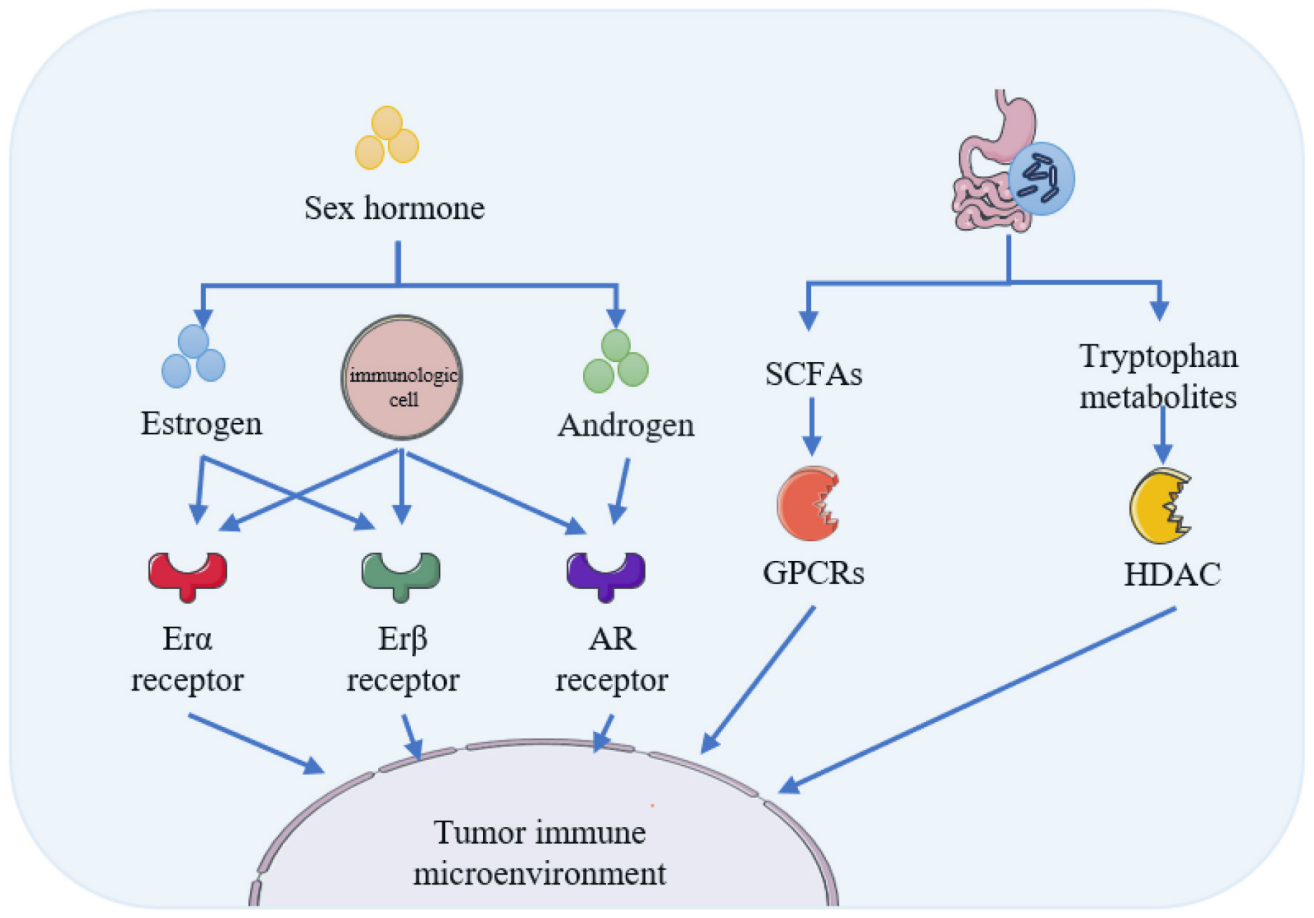
Immunoregulatory and inflammatory pathways between the gut microbiota and sex hormones.

Sex hormones not only directly regulate the function and activity of the immune system but also indirectly shape the structure and function of the gut microbiota by modulating intestinal immunity. Concurrently, the gut microbiota, through its metabolites and component antigens, provides feedback that fine-tunes immune responses, thereby forming a complex tripartite network involving sex hormones, gut microbiota, and the immune system. The schematic diagram depicting the mechanisms underlying immune and inflammatory processes is presented in Figure 2.

Both innate and adaptive immune responses are subject to sexual dimorphism (Klein and Flanagan, 2016). Nearly all immune cells express sex hormone receptors (Ozdemir and Dotto, 2019), and the promoter regions of many immune-related genes contain response elements for androgen receptor (AR) (Mantalaris et al., 2001) and estrogen receptor (ER) (Pierdominici et al., 2010). Research indicates that the anti-inflammatory effects of androgens, together with the context-dependent pro- and anti-inflammatory roles of estrogens, help explain the differential impact of sex hormones on innate and adaptive immunity (Papagerakis et al., 2022). Specifically, estrogen upregulates the expression of Toll-like receptors (TLRs) and pro-inflammatory factors via ERα to promote inflammatory responses, while it suppresses inflammation through ERβ. Correspondingly, the anti-inflammatory effects of androgens may be mediated either through direct AR-dependent mechanisms or via local conversion of androgens to estrogens (Nelson and Lenz, 2017).

These hormone-shaped immune landscapes serve as a key driver of sexual dimorphism in the gut microbiota. Such differences manifest at multiple levels. First, in overall community structure, the microbial composition of female mice more closely resembles that of pre-pubertal or castrated males than sexually mature males (Bernstein et al., 2023). Second, in terms of diversity, male mice generally exhibit lower microbial species richness and evenness compared to females of the same age (Bernstein et al., 2023). Furthermore, both animal and human studies confirm that certain bacterial taxa consistently show sex-specific enrichment, with higher abundance in one sex over the other (Brettle et al., 2022).

On the other hand, gut microbes can influence the differentiation and function of immune cells through the production of bioactive molecules such as short-chain fatty acids (SCFAs), tryptophan metabolites, and bile acids. SCFAs transmit anti-inflammatory signals via G protein-coupled receptors (GPCRs), including GPR41 (Yang et al., 2020), GPR43 (Koh et al., 2016), and GPR109a (Dalile et al., 2019), and act as histone deacetylase (HDAC) inhibitors. These actions promote IL-22 production by CD4+ T cells and innate lymphoid cells, thereby enhancing the intestinal mucosal barrier and alleviating inflammatory responses (Yang et al., 2020). Tryptophan metabolites play a crucial role in maintaining intestinal immune tolerance and microbial homeostasis (Gao et al., 2018). Metabolites such as indole and its derivatives help regulate intestinal barrier integrity and immune cell function by activating the pregnane X receptor (PXR) or the aryl hydrocarbon receptor (AhR) (Zelante et al., 2013). The specific mechanisms by which bile acids influence immune cells will be further elaborated in Section 2.3 on metabolic regulation.

### 2.3 Metabolic regulation

**Figure 3:**
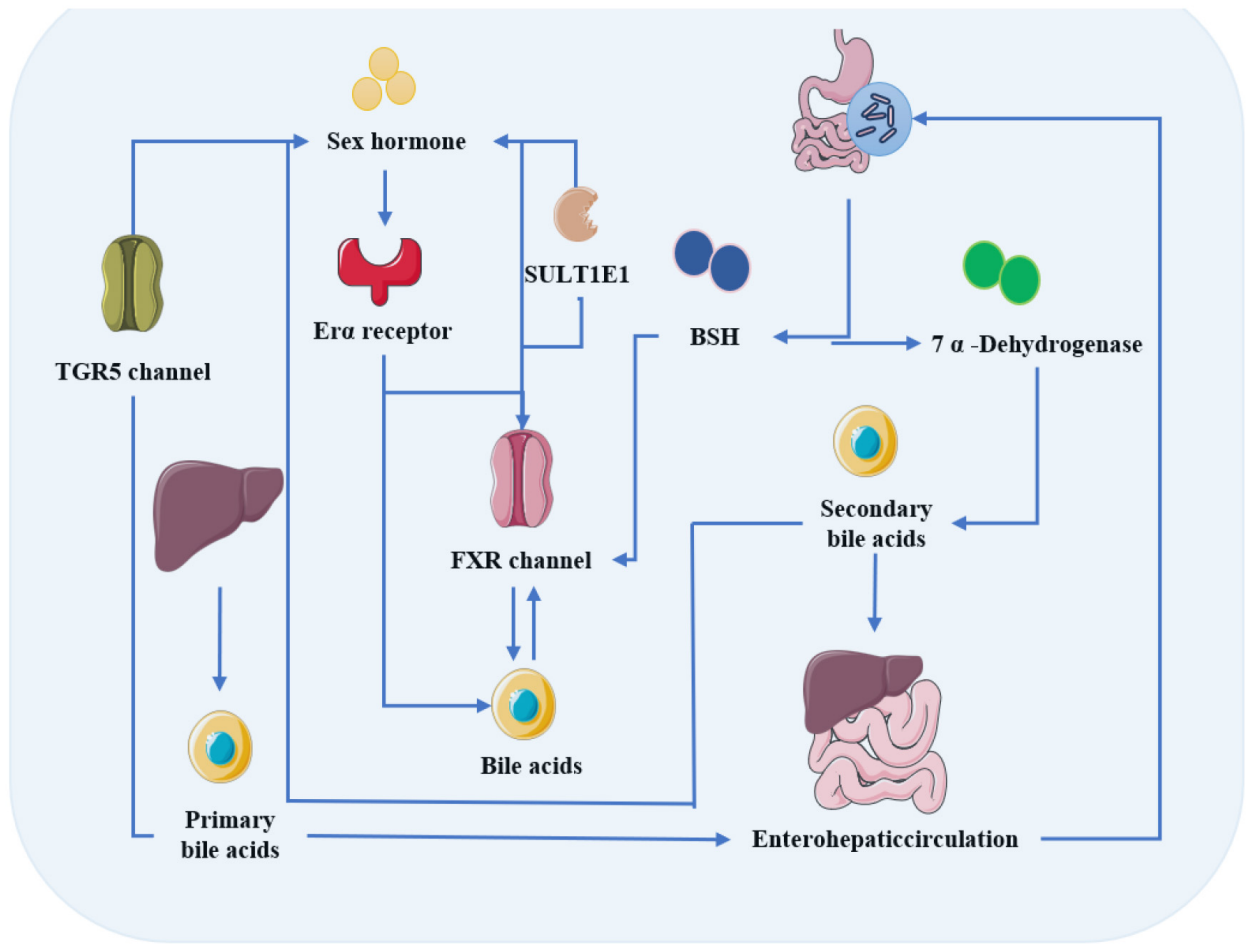
Metabolic regulation between the gut microbiota and sex hormones.

Sex hormones can directly regulate the synthesis, metabolism, and enterohepatic circulation of bile acids. Sex hormones can also indirectly shape the structure and function of the gut microbiota by modulating the bile acid profile (Larabi et al., 2023). Concurrently, the gut microbiota directly participates in the metabolic transformation of bile acids through its complex enzymatic systems such as bile salt hydrolase (BSH) and 7α-dehydroxylase, thereby establishing an intricate negative feedback regulatory network (Islam et al., 2011; Winston and Theriot, 2020). The metabolism of bile acids, along with the substrates and products of the key enzymes involved, is presented in Tables 1 and 2. A schematic diagram illustrating the mechanisms of metabolic regulation underlying the interplay between the gut microbiota and sex hormones is provided in Figure 3.

**Table 1:** Key bile acid pathways and their effects.

Metabolic pathway	Key players	Effects on sex hormones	Effects on the gut microbiome
Regulation of bile acid synthesis and transport	ERα, FXR	-	Estradiol-activated ERα can suppress FXR function, indirectly affecting hormone metabolism.
Modification of bile acids by gut microbiota	BSH	1. BSH activity inhibits the FXR pathway. 2. Increase BSH-active bacteria to improve dysbiosis.	
	HSDH/Dehydroxylase	Exogenous bile acids undergo 7α-dehydroxylation, promoting the proliferation of specific bacteria and simplifying microbiota composition.	
Direct regulation of sex hormones by bile acids	Ovarian granulosa cells (FXR pathway)		Decreases progesterone and estradiol levels; inhibits follicular development.
	TGR5 pathway		Promotes progesterone synthesis.
	Apoptosis induction		Reduces steroid hormone secretion.
	Hepatic metabolism (FXR Pathway)		Elevated estrogen levels

**Table 2:** Effects of probiotics on sex hormone imbalance-related disorders.

Disease	Species	Bacterial	Dosage (CFU)	Therapeutic effect	References
PCOS	*B. longum*	BL21	10^9^	HPA axis, metabolic regulation, Immunoregulatory and inflammatory pathways	Dong et al., 2025a
	*B. lactis*	V9	10^10.6^	Composition of gut microbiota	Zhang et al., 2019
	*L. plantarum*	CCFM1019	10^9^	Microbiome-gut-brain axis	He et al., 2022
	*L. paracasei*	DSM 27449	10^9^	Improve ovarian function, Reduce cystic follicle count	Lee et al., 2024
	AKK		**-**	Strengthen the intestinal barrier	Chiantera et al., 2023
EMs	*L. acidophilus*		10^6^	Immunoregulatory and inflammatory pathways, Composition of gut microbiota	Baker et al., 2017; Mehdizadeh et al., 2022
	*L. gasseri*	OLL2809	5 × 10^8^	Immunoregulatory and inflammatory pathways	Uchida and Kobayashi, 2013; Itoh et al., 2011
	*L. plantarum, fermentum, gasseri*		10^9^	Composition of gut microbiota	Baker et al., 2017
BC	*L. plantarum*		2 × 10^10^	Apoptosis	Budu et al., 2024
	*Saccharomyces boulardii*		1,500 μg/mL	Apoptosis	Pakbin et al., 2022
	*B. longum, L. acidophilus, Enterococcus faecalis*		2 × 10^8^, 2.7 × 10^8^, 2.5 × 10^9^	Composition of gut microbiota, Immunoregulatory and inflammatory pathways	Maroof et al., 2012; Imani et al., 2015; Juan et al., 2021; Marschalek et al., 2017
	CB, AKK		1.5 × 10^8^, 1.5 × 10^9^	Immunoregulatory and inflammatory pathways, Apoptosis	Li et al., 2025
	EcN	1917	10^9^	Immunoregulatory and inflammatory pathways	Shi et al., 2019
	*L. reuteri*		10^8^	Immunoregulatory and inflammatory pathways	Sajjad et al., 2024
	*L. casei*	CRL431	10^9^	Immunoregulatory and inflammatory pathways	Mendez et al., 2021
	*L. casei*	Shirota	**-**	Immunoregulatory and inflammatory pathways, Direct interactions	Kaga et al., 2013
EC	*Bifidobacterium, Lactobacillus*		10^10^	Direct interactions	Chen et al., 2025
	Sodium butyrate		**-**	Cell cycle arrest, DNA damage, Oxidative stress, Apoptosis, Direct interactions	Yu et al., 2014; Kato et al., 2011; Saito et al., 1991; Terao et al., 2001; Zang et al., 2019
Cervical cancer	*Lactobacillus*		10^9^	Direct interactions	Pawar and Aranha, 2022
	*L. casei*	SR1, SR2	10^7^	Apoptosis	Riaz et al., 2018
	*L. paracase*	SR4	10^7^	Apoptosis	Riaz et al., 2018
	*L. casei*	LH23	10^9^	Direct interactions, Apoptosis	Steinholtz, 1986
	*L. casei*	TD-2	10^9^	Immunoregulatory and inflammatory pathways	Abdolalipour et al., 2022
	*L. crispatus, jensenii, gasseri*		1.5 × 10^10^	Cell cycle arrest	Wang et al., 2018
	*L. plantarum*		10^7^	Direct interactions	Chuah et al., 2019; Nami et al., 2014
	*L. fermentum*	Ab.RS22	**-**	Apoptosis, Direct interactions	Asoudeh-Fard et al., 2024
	*L. fermentum*	CH, KH	4.8 × 10^8^	Apoptosis, Direct interactions	Asoudeh-Fard et al., 2024
	*B. adolescentis*	SPM1005-A	5.1 × 10^7^	Direct interactions	Cha et al., 2012
PCa	*L. acidophilus*	La-05, La-03	10^8^	Apoptosis, Direct interactions	Rosa et al., 2020
	*L. casei*	01	10^9^	Apoptosis, Direct interactions	Rosa et al., 2020
	*Bifidobacterium*	Bb-12	10^8^	Apoptosis, Direct interactions	Rosa et al., 2020
	LGG		**-**	Direct interactions	Celebioglu, 2021
	*L. reuteri*		10^8^-10^9^	Immunoregulatory and inflammatory pathways	Nascimento et al., 2014
ED	*L. rhamnosus, plantarum*		10^9^	Direct interactions, HPA axis	Edem et al., 2021; Wu et al., 2024a
	*B. longum*	BL21	10^10^	Direct interactions	Dong et al., 2025b
	*B. longum*	B8762	10^9^	Direct interactions	Zhao et al., 2025
	LGG		10^9^-10^10^	Direct interactions, oxidative stress, Immunoregulatory and inflammatory pathways	Guo et al., 2020
	*L. rhamnosus*	CECT8361	2–4 × 10^9^	Direct interactions	Bozorgpoursavadjani et al., 2025; Valcarce et al., 2019
	*L. brevis*	GKJOY	**-**	Oxidative stress, Immunoregulatory and inflammatory pathways	Hwang et al., 2025
	*L. mesenteroides*	SD23	10^10^	Immunoregulatory and inflammatory pathways	Castro-Rodriguez et al., 2020
	LGG	NCDC-610	4 × 10^9^	HPA axis	Akram et al., 2023
	*L. fermentum*	NCDC-40	4 × 10^9^	HPA axis	Akram et al., 2023
	*Levilactobacillus*	505	10^7^	HPA axis	Joung et al., 2022

All probiotics are administered orally.

Sex hormones primarily regulate bile acid metabolism via nuclear receptor signaling pathways. Studies have shown that ERα suppresses the expression of bile acid and cholesterol transport proteins in the liver (Yamamoto et al., 2006). The nuclear receptor farnesoid X receptor (FXR) also plays a critical role in modulating bile acid metabolism. Research has shown that the FXR agonist chenodeoxycholic acid (CDCA) upregulates the expression of the bile salt export pump (BSEP), thereby promoting bile acid transport and reducing systemic bile acid levels (Zou et al., 2008). Furthermore, ERα can interact with FXR in an estradiol-dependent manner and inhibit its function *in vitro*, contributing to the regulation of bile acid metabolism (Milona et al., 2010). ERα also suppresses FXR-mediated signaling, leading to alterations in bile acid composition and distribution, and ultimately disrupting bile acid homeostasis (Zhao et al., 2023).

The interplay between bile acids and the gut microbiota is complex. This interaction plays a significant role in shaping the composition of microbial community (Larabi et al., 2023). Due to their lipophilic properties, bile acids can exert direct antibacterial effects by targeting bacterial membranes (Begley et al., 2005). Bile acids also activate several receptor signaling pathways, including FXR and the G protein-coupled bile acid receptor 1 (Inagaki et al., 2006; Maruyama et al., 2006). Research has found that the bile acid analog obeticholic acid, an FXR agonist, inhibits endogenous bile acid synthesis and promotes the proliferation of Gram-positive bacteria such as *Streptococcus thermophilus* and *Lactobacillus casei* (*L. case*i) (Farese et al., 1982).

The gut microbiota modifies the structure and hydrophobicity of bile acids within the enterohepatic circulation, thereby enhancing bile acid diversity (Poland and Flynn, 2021). Key metabolic pathways include the deconjugation reaction mediated by BSH, as well as various modifications catalyzed by hydroxysteroid dehydrogenases (HSDHs) or dehydroxylases, which generate secondary bile acids (Song et al., 2020). Among these, the deconjugation of taurine and glycine-conjugated bile acids serves as the primary step for all subsequent modifications. This process cleaves glycine or taurine residues, releasing unconjugated primary bile acids (Larabi et al., 2023). Studies indicate that *Bacteroides fragilis* can suppress FXR signaling through its BSH activity, thereby modulating bile acid metabolism (Tang et al., 2023). Similarly, the Jiang-Tang-San-Huang pill has been shown to ameliorate gut dysbiosis by enriching BSH-active bacteria (such as *Bacteroides, Lactobacillus*, and *Bifidobacterium*), promoting bile acid accumulation, and alleviating type 2 diabetes (Wu et al., 2023). Additionally, exogenous bile acids can be efficiently converted into deoxycholic acid via bacterial 7α-dehydroxylation. This process simplifies microbial community composition and promotes the proliferation of *Clostridia* and *Erysipelotrichia* (Islam et al., 2011).

Bile acids regulate sex hormones through multiple mechanisms, primarily involving the FXR pathway. Locally in the ovary, bile acids activate the FXR signaling pathway in granulosa cells, suppressing the expression of steroidogenic genes such as StAR, CYP11A1, and CYP19A1. This results in reduced progesterone and estradiol levels and impaired follicular development (Zhu et al., 2025). In contrast, conjugated bile acids such as CDCA upregulate the expression of StAR and CYP11A1 via Takeda G protein-coupled receptor 5 (TGR5), thereby promoting progesterone synthesis (Chen M. et al., 2024). Moreover, glycine-conjugated deoxycholic acid (GDCA) induces granulosa cell apoptosis, further diminishing steroid hormone secretion (Wei et al., 2022). Systemically, bile acids—particularly under cholestatic conditions—inhibit sulfotransferase family 1E member 1 (SULT1E1) via the FXR pathway, impairing hepatic estrogen clearance and ultimately leading to abnormally elevated systemic estrogen levels (Liu et al., 2018).

### 2.4 Microbiome-gut-brain axis regulation

**Figure 4:**
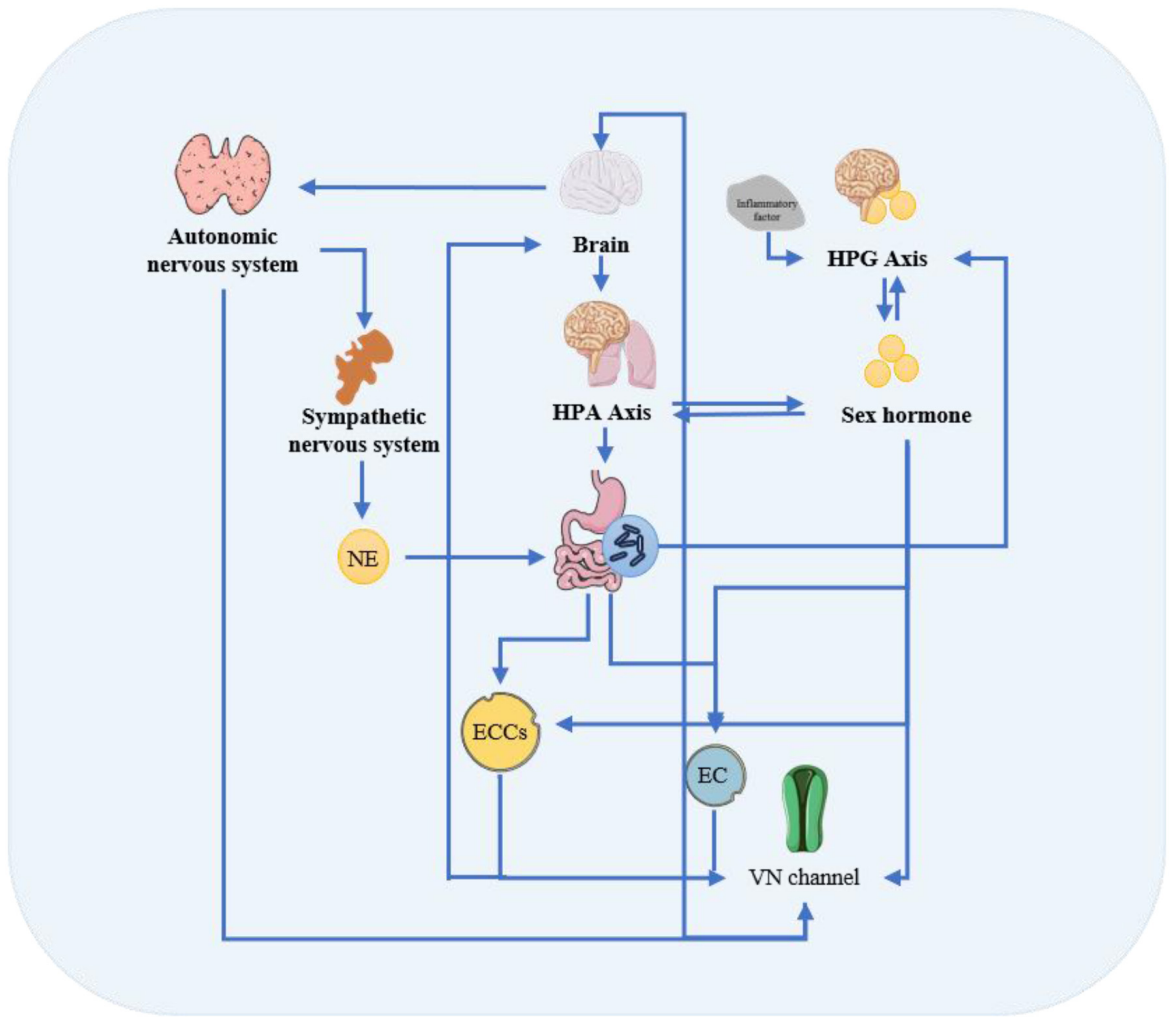
Microbiome-gut-brain axis.

MGBA constitutes a complex bidirectional communication network involving neural, endocrine, and immune pathways, serving as a critical interface for interactions between the gut microbiota and sex hormones (Mazzoli and Pessione, 2016). Key MGBA pathways that have garnered research focus include the autonomic nervous system (ANS), the hypothalamic-pituitary-gonadal (HPG) axis, the hypothalamic–pituitary–adrenal (HPA) axis, and enteroendocrine cells (EECs) (1965; Cryan et al., 2019). Presented in Figure 4 is a schematic diagram illustrating the mechanisms underlying vagal pathways and autonomic regulation, the HPA/HPG axis, as well as enteroendocrine cell-mediated neurotransmitter signaling.

#### 2.4.1 Vagal pathways and autonomic regulation

The ANS, comprising sympathetic and parasympathetic branches, forms a foundational link between the HPG and HPA axis (Longo et al., 2023). The vagus nerve (VN), a major component of the parasympathetic ANS, represents the most rapid and direct pathway connecting the gut and the brain (Berthoud and Neuhuber, 2000). Binge eating has been shown to induce markable alterations in the gut microbiota, suppress activity in intestinal vagal terminals, and subsequently hyperactivate the vagus–nucleus tractus solitarius–paraventricular thalamus–gut–brain circuit (Fan et al., 2023). Concurrently, gut microbiota dysbiosis can impair adult hippocampal neurogenesis and provoke depression-like behaviors (Siopi et al., 2023). Serotonin acts as a pivotal mediator within the MGBA; approximately 90% of 5-hydroxytryptamine (5-HT) is produced by enterochromaffin cells (ECs) in the gastrointestinal epithelium (Israelyan and Margolis, 2018), and the production of both 5-HT and dopamine exerts significant influence on early brain development (Wang et al., 2020). Studies indicate that gut microbial metabolites can enhance dopamine release from the ventral tegmental area to the nucleus accumbens by activating vagal afferent fibers, thereby promoting euphoric sensations. Notably, certain bacterial strains (e.g., *Enterobacte*r) are capable of directly synthesizing 5-HT, suggesting that gut microbes may participate in the regulation of social behavior and emotional responses via the gut–brain axis (Hwang and Oh, 2025). Meanwhile, sex hormones can induce axonal growth in diverse neuronal systems and modulate the development of brain circuitry (Cao et al., 2014). Research has demonstrated that androgens enhance airway responsiveness to cholinergic stimulation in mice through a vagally mediated reflex mechanism (Card et al., 2007), while circulating estrogen levels in females not only alter the function of afferent and efferent vagal neurons but also modulate the activity of autonomic-regulating neurons in the brainstem that receive vagal inputs (Ciriello and Caverson, 2016).

#### 2.4.2 HPA/HPG axis

The brain has been shown to regulate both gut microbiota composition and sex hormone levels through multiple mechanisms. Under the stress, the brain activates the sympathetic nervous system and inhibits the parasympathetic nervous system, releasing neurotransmitters and neuromodulators to the gut, thereby substantially altering gut microbial structure and inducing intestinal inflammatory responses (Wang et al., 2020). This process further suppresses gonadotropin-releasing hormone (GnRH) releasing from the hypothalamus via other MGBA pathways, leading to inhibition of HPG axis function and a decline in sex hormone levels (Trotman et al., 2013). Specifically, stress stimuli activate the intestinal sympathetic nervous system to release norepinephrine (NE) (Zhang et al., 2023). NE has been shown to bind to certain pathogenic bacteria and enhance their virulence. Concurrently, alterations in serotonin and corticotropin-releasing factor/hormone (CRF/CRH) signaling that was associated with depression lead to changes in intestinal motility, increased fluid secretion, and elevated gut permeability, thereby contributing to dysbiosis (Hoogendoorn et al., 2017). Moreover, stress-induced elevations in glucocorticoids inhibit reproductive function of the HPG axis, primarily by suppressing GnRH release at the hypothalamic level (Whirledge and Cidlowski, 2010). The ANS also modulates a range of fundamental gastrointestinal functions, thereby shaping the ecological niche of the gut microbiota (Wang S. Z. et al., 2020; Zhao et al., 2025). Research has demonstrated that an acidic environment not only promotes the growth of beneficial bacteria such as *Lactobacillus* and *Bifidobacterium* but also enhances the competitive fitness of acidophilic strains (Atasoy et al., 2024). This phenomenon may be attributed to the acid-facilitated production of short-chain fatty acids by probiotics, which further lowers the environmental pH, thereby establishing a positive feedback loop (Du et al., 2023). In contrast, alkaline conditions favor the proliferation of opportunistic pathogens, including enterotoxigenic *Escherichia coli* and *Vibrio cholera* (Gonzales-Siles et al., 2017). Notably, when the pH reaches 6.5, the growth of the conditionally pathogenic genus *Bacteroides* is promoted. This is likely due to the peak production of propionate at this pH level, a metabolite associated with *Bacteroides* metabolism, which in turn creates a favorable niche for its expansion (Walker et al., 2005). Dysregulation of the ANS can lead to gut microbiota disruption, compromise its immunity, and trigger systemic chronic low-grade inflammation (Aghara et al., 2025). In this context, pro-inflammatory cytokines may further inhibit the synthesis and secretion of sex hormones by acting on the gonads or suppressing GnRH release (Jin et al., 2021).

As a component of the MGBA, chronic activation of the HPA axis has been confirmed to influence gut microbiota composition and intestinal permeability (Yoon and Kim, 2021). The underlying mechanisms may involve increased gut barrier permeability and a microbiota-driven pro-inflammatory state (de Punder and Pruimboom, 2015). Studies indicate that probiotics such as *Lactobacillus* and *Bifidobacterium* can alleviate stress-induced HPA axis dysfunction and depression/anxiety-like symptoms (Misiak et al., 2020), and improve learning and memory capacity. Mechanistically, stress-induced activation of the HPA axis triggers the release of corticotropin-releasing hormone, adrenocorticotropic hormone (ACTH), and cortisol, which further affect gut function by inhibiting microbial growth and altering intestinal motility (Quiroga-Castaneda et al., 2024). Additionally, changes in gonadal steroid levels regulated by the HPG axis are involved in this process. Estrogen and androgens modulate HPA axis activity through their receptors (Handa and Weiser, 2014). It is noteworthy that androgens exert significant organizational effects on the HPA axis during early development, potentially mediated either through direct binding of testosterone to the androgen receptor or indirectly via aromatization of testosterone to estradiol (Seale et al., 2005).

#### 2.4.3 Enteroendocrine cells and neurotransmitter signaling

EECs respond to sex steroids and gut microbial metabolites, influencing both gastrointestinal physiology and central nervous system function through multiple signaling pathways (Hokanson et al., 2024). EECs can act indirectly by releasing peptides, hormones, and neurotransmitters, or directly modulate vagal and central neural activity via excitatory synaptic connections (Kaelberer et al., 2018). Research has found that *Edwardsiella tarda* activates EECs through the transient receptor potential ion channel Trpa1 and promotes intestinal motility (Ye et al., 2021). Meanwhile, hormones and small-molecule neurotransmitters secreted by EECs can activate extrinsic vagal afferent nerves, regulating central processes such as appetite and food preference (Davison et al., 2025). Regarding hormonal regulation, sex steroids including estrogen and androgens modulate hormone secretion, gene expression, and cell fate in EECs via nuclear and membrane receptors. Specifically, estrogen upregulates the synthesis and secretion of glucagon-like peptide-1 (GLP-1) in intestinal L-cells through ERβ (Handgraaf et al., 2018), and stimulates cholecystokinin (CCK) secretion from I-cells via ERα (Peng et al., 2023). Furthermore, the expression of tryptophan hydroxylase 1 (TPH1), the rate-limiting enzyme in 5-HT synthesis in ECs, is positively regulated by estrogen (Wang et al., 2025).

### 2.5 The impact of species differences on the gut microbiota-sex hormone axis

Although mice and rats remain the most commonly used animal models for investigating the interplay between gut microbiota and sex hormone regulation, substantial differences exist between these rodents and humans. Such differences are evident in gut microbial composition, sex hormone metabolic pathways, and receptor distribution, which may directly influence the mechanisms underlying sex hormone modulation. For instance, at the phylum level, the gut microbiota composition of mice and rats resembles that of humans; however, marked distinctions emerge at the genus level (Poo et al., 2024). Genera such as *Prevotella, Faecalibacterium*, and *Ruminococcus* are more abundant in the human gut microbiota, whereas *Lactobacillus, Alistipes*, and *Turicibacter* are enriched in the murine intestine. In contrast, *Clostridium, Bacteroides*, and *Blautia* exhibit comparable relative abundances between the two species (Tokuhara, 2021). Beyond species-specific differences in microbial composition, fundamental discrepancies also exist in the synthesis and metabolism of sex hormones. First, with regard to hormone levels and fluctuation patterns, the estrous cycle of mice lasts approximately 4–5 days, whereas the human menstrual cycle spans about 28 days, during which estradiol and progesterone exhibit cyclic variations—resulting in markedly distinct fluctuation profiles between species (Shadani et al., 2024; Chandimali et al., 2026; Hersey et al., 2023). Second, humans and mice differ in the expression of key metabolic enzymes such as GUS. Moreover, the tissue distribution of ERα and ERβ, both of which are co-expressed across species, displays species-specific patterns (Kirilenko et al., 2019). For instance, both ERα and Erβ are expressed in mammalian ovarian tissues. These two receptors are also detectable in the brain and lung. Of note, ERβ is barely expressed in the mammary gland tissue of female mice or in the testicular cells of male mice. In contrast, in rats, ERα exhibits moderate to high expression levels in the uterus and testes, whereas ERβ shows the strongest expression in the prostate and ovaries (Couse et al., 1997). These interspecies discrepancies may directly lead to divergent outcomes of the same intervention on the gut microbiota–sex hormone axis. Therefore, during the clinical trial phase, priority should be given to validating findings using human-derived microbiota data and clinical samples, with animal experiments serving as complementary evidence. Furthermore, clinical trial designs must adequately account for confounding factors such as fluctuations in the sex hormone cycle, menopausal status, and the use of hormone-based medications. Standardized methodologies for microbiota analysis and hormone measurement should also be implemented.

## 3 Mechanisms of probiotic intervention in sex hormone-related disorders

### 3.1 Polycystic ovary syndrome

PCOS represents the most prevalent endocrine and metabolic disorder among women of reproductive age, clinically characterized by hyperandrogenism, ovulatory dysfunction, and polycystic ovarian morphology (Di Lorenzo et al., 2023). Currently, there remains no effective therapy for PCOS, with clinical management primarily relying on combined approaches such as oral contraceptives, insulin sensitizers, cyclic progestins, or anti-androgen agents (Lei et al., 2020). Recent evidence has established a close association between gut microbiota dysbiosis and PCOS pathogenesis. With their safety and effectiveness functions, probiotics have become promising therapeutic avenue (Salehi et al., 2024).

Probiotics ameliorate endocrine disturbances in PCOS through multiple mechanisms. First, probiotics modulate gut–brain axis signaling. Studies demonstrate that *Bifidobacterium longum* (*B. longum*) subsp. longum BL21 alleviates dihydrotestosterone (DHT)-induced PCOS via the gut–brain–ovary axis, improving metabolic parameters, attenuating inflammatory responses, and exerting neuroprotective effects (Dong et al., 2025a). The butyrate-dependent pathway constitutes a key component of gut–brain communication. *L. plantarum* CCFM1019 enhances butyrate and peptide YY levels through GPR41 receptor modulation, thereby ameliorating letrozole-induced PCOS in rats (He et al., 2022). Furthermore, *L. paracasei* subsp and *L. paracasei* DSM 27449 has been shown to improve ovarian function, reduce cystic follicle count, and lower serum testosterone levels (Lee et al., 2024).

Second, probiotics directly regulate sex hormone levels by influencing hormonal metabolism. For instance, *B. lactis* V9 modulates circulating sex hormone concentrations in PCOS patients through gut microbiota remodeling (Zhang et al., 2019). Other *lactobacilli* have also been found to alleviate PCOS by regulating gut microbial communities involved in steroid hormone metabolism (He et al., 2020).

Insulin resistance constitutes a core pathological feature of PCOS, and probiotics enhance insulin sensitivity through diverse pathways. Metformin, a widely prescribed insulin sensitizer, demonstrates superior efficacy in regulating insulin levels, improving glycemic control and insulin resistance, and modulating gut microbiota composition when co-administered with probiotics (Li and Peng, 2025; Luo et al., 2025). Additionally, combined intervention with probiotics and vitamin D has been confirmed to improve insulin function and TNF-α gene expression (Banikazemi et al., 2025; Ostadmohammadi et al., 2019).

Chronic inflammation underlies PCOS pathology, and probiotics exert anti-inflammatory effects through multiple mechanisms. Evidence indicates that probiotics mitigate hypothalamic lipid accumulation, suppress inflammatory responses, and enhance antioxidant capacity, thereby significantly alleviating PCOS-associated chronic inflammation (Areloegbe et al., 2025). Concurrently, specific probiotics such as *Akkermansia muciniphila* (AKK) reinforce intestinal barrier function by promoting mucus layer integrity and maintaining gut mucosal homeostasis, reducing the host inflammation (Chiantera et al., 2023).

### 3.2 Endometriosis

Endometriosis (EMs), also referred to as secondary dysmenorrhea, is a chronic inflammatory condition characterized by estrogen dependence and clinically manifested by intense cramping, chronicpelvic pain, infertility, and menstrual abnormalities (Giudice and Kao, 2004). This disease is pathologically defined by the presence and proliferation of endometrial tissue outside the uterine cavity and myometrium, accompanied by chronic inflammatory responses caused by endometrial tissue growth and infiltration (Jiang et al., 2021). Emerging evidence indicates a close association between gut microbiota dysbiosis and EMs. Probiotics, as bioactive beneficial microorganisms, offer novel therapeutic perspectives for EMs through multiple mechanisms involving microbial ecological balance restoration, inflammation modulation, and immune regulation.

Gonadotropin-releasing hormone agonists (GnRHa) represent a first-line pharmacological intervention for EMs, acting through stimulation of follicle-stimulating hormone (FSH) and luteinizing hormone (LH) production to suppress estrogen synthesis, thereby achieving therapeutic effects (Khan et al., 2016). This suggests that stability of estrogen metabolism is intimately linked to endometriosis risk, and appropriate regulation of estrogen metabolism may help prevent disease onset and progression (Zervou et al., 2023). Research demonstrates that probiotics expressing GUS activity can modulate estrogen levels in menopausal transition women (Honda et al., 2024), while elevated enzymatic activity of this kind influences the number and volume of endometriotic lesions as well as macrophage infiltration (Wei et al., 2023).

In EMs, the peritoneal microenvironment exhibits chronic inflammation with infiltration of immunologically aberrant immune cells, leading to systemic immune dysregulation and creating an ideal niche for disease progression (Symons et al., 2018). Studies reveal that *L. acidophilus* functions as an antigenic compound that induces interleukin-1 (IL-1) and interleukin-6 (IL-6) secretion but reduce prototypical Th2 cytokine secretion, differentially stimulating Th1-type immune responses to exert therapeutic effects (Mehdizadeh et al., 2022). Additionally, *L. gasseri* OLL2809 suppresses EMs development by activating natural killer (NK) cell (Itoh et al., 2011; Uchida and Kobayashi, 2013). Clinical investigations further demonstrate that interventions by administration with *L. acidophilus, L. plantarum, L. fermentum*, and *L. gasseri* significantly improve Visual Analog Scale (VAS) scores for pain—a tool widely employed in the assessment of cancer-related pain, neuropathic pain, and related clinical conditions (Khodaverdi et al., 2019). Additional evidence suggests that maintaining microbial homeostasis may help inhibit ectopic endometrial tissue overgrowth and hyperproliferation (Baker et al., 2017).

### 3.3 Sex hormone-related tumors

#### 3.3.1 Breast cancer

Breast cancer (BC) represents the most frequently diagnosed malignancy in women and ranks as the second most common cancer globally (Evans and Howell, 2007). Despite advancements in early detection and multimodal therapeutic strategies that have improved patient prognosis, BC management remains a formidable challenge due to tumor heterogeneity, drug resistance, and immune dysfunction (Liang et al., 2020). Recent microbiome research has provided novel perspectives on BC treatment. Evidence indicates that probiotics affect BC initiation and progression through diverse pathways including immunomodulation, metabolite production, and inflammatory control.

Apoptosis, a form of programmed cell death, plays a central role in eliminating abnormal cells such as cancer cells. Specific probiotic strains or their metabolites can induce apoptosis or cell death in BC cells. For instance, *L. plantarum* (Budu et al., 2024) and *Saccharomyces boulardii* supernatant (Pakbin et al., 2022) alleviate breast carcinogenesis by inducing apoptosis in A375 and MCF-7 BC cell lines. Beyond apoptosis induction, probiotics can interfere with cell cycle progression and suppress invasive and metastatic capabilities of BC cells. Research demonstrates that metabolites from GABA-producing *Limosilactobacillus fermentum* inhibit MCF-7 cell migration, downregulate gene and protein expression of matrix metalloproteinases (MMP-2, MMP-9), and induce cell cycle arrest at the G2/M phase (Ngo et al., 2025).

Probiotics maintain gut microbial equilibrium through competitive exclusion of pathogens and production of antimicrobial substances and organic acids. Significant differences in gut microbiota composition have been identified between BC patients and healthy individuals (Nandi et al., 2023). Probiotics can adjust gut microbial communities and improve metabolic and anthropometric parameters (Pellegrini et al., 2020). Administration of probiotic supplement during docetaxel-based chemotherapy may mitigate weight gain, reduce increases in body fat percentage and plasma LDL, and minimize metabolic alterations and gut dysbiosis (Juan et al., 2021). Furthermore, oral administration of *Lactobacillus* alone may improve vaginal microbiota in women undergoing BC chemotherapy (Marschalek et al., 2017).

Probiotics can also modulate the host immune system to establish an enhanced “immune surveillance” environment that is unfavorable for tumor initiation and progression. Specifically, oral administration of *Clostridium butyricum* (CB) and AKK inhibits 4T1 BC progression, with combined treatment (CB-AKK) demonstrating significantly superior efficacy compared to individual strains. The CB-AKK combination activates antitumor immunity in mice, remodels the tumor microenvironment, and suppresses BC cell proliferation while promoting tumor apoptosis via Bcl-2/Bax signaling pathway activation (Li et al., 2025). T cells play a central role in the host immune system. For instance, T helper type 1 (Th1) cells facilitate the activation of CD8^+^ T cells and macrophages, thereby enhancing antitumor immunity. In contrast, regulatory T cells (Tregs) exert immunosuppressive effects that, while preventing excessive autoimmune reactions, can also inhibit tumor immune responses (Luo et al., 2019). Probiotics contribute to immune regulation by promoting a more pronounced Th1-biased response, which strengthens targeted tumor clearance—while reducing the number or function of immunosuppressive Tregs within the tumor milieu (Jing et al., 2025). Studies indicate that *L. acidophilus* promotes Th1-biased immune responses and may enhance antitumor immunity (Imani et al., 2015; Maroof et al., 2012). *Escherichia coli strain Nissle* 1917 (EcN) alleviates immunosuppressive tumor microenvironments through enhanced tumor-specific effector T-cell infiltration and dendritic cell activation (Shi et al., 2019). Additionally, *L. reuteri* (Sajjad et al., 2024) and *L. casei* CRL431 (Mendez et al., 2021) exhibit chemoprotective and immunomodulatory potential against cadmium chloride-induced BC in mice.

Chronic inflammation provides a fertile ground for cancer initiation and progression. Probiotics significantly reduce pro-inflammatory cytokines (e.g., TNF-α, IL-6, IFN-γ) while promoting anti-inflammatory factor (e.g., IL-10) expression. This immunomodulatory effect, mediated through the gut-immune axis, exerts a protective influence on mammary tissue and contributes to the suppression of BC initiation. For example, *L. plantarum* enriched with selenium nanoparticles (SeNP) effectively induces immune responses by suppressing pro-inflammatory cytokines including IFN-γ, TNF-α, and IL-2 while enhancing NK cell activity (Yazdi et al., 2012).

Circulating estrogen has been established as a significant biomarker in BC, contributing to enhanced cancer cell proliferation, angiogenesis, metastatic stimulation, and chemotherapy resistance (Muccee et al., 2022). Probiotics selectively reduce viability of estrogen receptor-positive (ER^+^) BC cells and alter mitochondrial metabolism in non-cancerous epithelial cells. Concurrently, tamoxifen modifies mammary tissue microbiota by increasing abundance of commensal *Lactobacillus* and *Streptococcus* species, suggesting that enhancing mammary probiotic populations may reduce tumor burden and improve disease-free survival (Abolhassani et al., 2025). Combined consumption of soy isoflavones with *L. casei* Shirota reduces BC risk. Soymilk combined with *L. casei* Shirota decreases ER-α-positive and Ki-67-positive tumor cells more effective compared to soymilk alone (Kaga et al., 2013).

#### 3.3.2 Endometrial cancer

Endometrial cancer (EC) ranks among the most common gynecological malignancies worldwide, with increasing incidence rates (Xu et al., 2022). Estrogen stimulate endometrium to oppose the progesterone-mediated differentiation, which represents primary etiological factor associated with endometrial hyperplasia and cancer development (Okuda et al., 2010). Although early-stage EC patients can achieve cure through surgery, advanced and recurrent cases generally exhibit poor prognosis, and current treatments (e.g., radiotherapy, chemotherapy) frequently involve adverse effects (Pu et al., 2020). As a complex endocrine and immunomodulatory system, the homeostasis of gut microbiota, plays crucial roles in disease pathogenesis. Restoring microbial homeostasis through probiotic supplementation offers novel approaches for comprehensive EC management.

Research indicates that probiotic intervention increases abundance of beneficial bacteria such as *Bifidobacterium* and *Lactobacillus*, while reducing levels of potentially harmful bacteria including *Bacteroidetes* and *Clostridium* (Chen et al., 2025). Furthermore, short-chain fatty acids, particularly butyrate produced through probiotic fermentation, function as potent HDAC inhibitors that reactivate tumor suppressor genes via epigenetic modifications, thereby inhibiting EC cell growth. Mechanistic studies reveal that sodium butyrate (SB) treatment increases estrogen receptor binding sites seven-fold in human endometrial adenocarcinoma (IK) cells and induces G1 phase cell cycle arrest, suppressing DNA synthesis without affecting overall RNA and protein levels (Saito et al., 1991). Further investigations demonstrate that this inhibition involves SB-mediated upregulation of p21 protein expression, leading to subsequent dephosphorylation of retinoblastoma protein (pRb) (Terao et al., 2001). Additionally, SB suppresses cancer cell growth through chromatin remodeling and gene expression regulation, and could become a promising targeted therapeutic agent for EC.

Another study found that SB significantly inhibits self-renewal capacity of endometrial cancer stem-like cells by inducing DNA damage and promoting reactive oxygen species (ROS) generation, while markedly increasing expression of DNA damage marker γH2AX, indicating heightened sensitivity of cancer cells to butyrate-induced damage (Kato et al., 2011). Moreover, SB promotes ferroptosis in EC cells by upregulating RBM3 expression and downregulating SLC7A11 (Wang et al., 2023). Regarding combination therapies, SB enhances doxorubicin cytotoxicity in uterine cancer cells by downregulating telomerase component hTERT expression and promoting apoptosis (Yu et al., 2014; Zang et al., 2019).

#### 3.3.3 Cervical cancer

Cervical cancer represents the fourth most common malignancy in women worldwide, primarily associated with persistent infection by high-risk human papillomavirus (HPV) (Wang et al., 2023). Despite significant advances in HPV vaccination and screening techniques, cervical cancer treatment, particularly for advanced-stage patients, continues to face challenges including recurrence, metastasis, and treatment-related side effects (Yuan et al., 2025). Current clinical management primarily involves surgery, radiotherapy, and chemotherapy, yet these approaches cannot prevent recurrence and may induce various adverse effects such as menstrual abnormalities and vaginal pain (Han et al., 2021).

A healthy vaginal environment dominated by *Lactobacillus* species constitutes the first line of defense against pathogenic infections (Parolin et al., 2021). Consequently, probiotic supplementation to restore and maintain healthy vaginal microbiota offers innovative approaches for comprehensive cervical cancer management. Studies demonstrate that *Lactobacillus* cell-free culture supernatants significantly upregulate E-cadherin expression in human cervical cancer cells (HeLa) and cervical squamous carcinoma cells (SiHa), while ELISA analyses reveal downregulation of matrix metalloproteinase-9 (MMP9) levels in HeLa cells, suggesting that *Lactobacillus*-derived metabolites may serve as biotherapeutic agents for controlling HPV infection and cervical cancer progression (Pawar and Aranha, 2022), with positive impacts on HPV clearance rates and cervical lesion regression in clinical practice (Susetiati et al., 2025).

*L. casei* SR1, SR2, and *L. paracasei* SR4 isolated from human breast milk exhibit substantial anticancer activity by upregulating pro-apoptotic genes (BAX, BAD, caspase-3, caspase-8, caspase-9) and downregulating anti-apoptotic gene BCl-2. SR1, SR2, and SR4 demonstrate significant HeLa cancer cell inhibition compared to controls (Riaz et al., 2018). Furthermore, *L. casei* LH23 suppresses HPV oncogene E6/E7 expression, thereby inhibiting cervical cancer cell proliferation, inducing apoptosis, slowing cell migration, and altering metastasis-related gene expression (Hu et al., 2023). Additional research indicates that *L. casei* TD-2 combined with granulocyte-macrophage colony-stimulating factor (GM-CSF) exerts stronger inhibitory effects on mouse lung epithelial cells (TC-1) than GM-CSF alone, while significantly elevating interferon-γ (IFN-γ), IL-4, and IL-12 levels, and increasing tumor necrosis factor-related apoptosis-inducing ligand (TRAIL) expression (Abdolalipour et al., 2022).

Supernatants from *Lactobacillus* including *crispatus, jensenii*, and *gasseri* modulate cell cycle progression in human cervical cancer intestinal metastasis cells (Caski cells), specifically reducing cyclin-dependent kinase-2 (CDK2) and cyclin A expression, increasing p21 expression, and accompanied by decreased E6 and E7 oncogene expression (Wang et al., 2018). Additionally, *L. crispatus* exhibits cytotoxic effects on HeLa cells (Nouri et al., 2016), while *L. crispatus* M247 attenuates cervical abnormalities induced by HPV infection by restoring physiological vaginal balance (Dellino et al., 2022).

*L. plantarum* demonstrates favorable probiotic properties and significant anticancer activity across multiple human cancer cell lines (including cervical cancer HeLa; gastric cancer AGS; colon cancer HT-29; breast cancer MCF-7), with no apparent toxicity to normal cells such as human umbilical vein endothelial cells (HUVEC) (Nami et al., 2014). This strain also inhibits HeLa cancer cell growth (Dwi et al., 2021), while its metabolites exert toxic effects on MCF-7 breast cancer cells through apoptotic mechanisms (Chuah et al., 2019). Further investigations revealed that its subspecies, Probio87, selectively inhibits *L. iners* without affecting *L. crispatus*, thereby demonstrating a favorable capacity for microecological regulation. Application of its cell-free supernatant significantly reduces proliferation and angiogenesis markers in cultured cervical cancer cells, induces apoptosis and cell cycle arrest in HPV-positive cells, with minimal effects on HPV-negative cervical cancer cells (C-33A) (Xu et al., 2025b).

Further investigations have demonstrated that the combination of *L. fermentum* with the chemotherapeutic agent vincristine sulfate promotes apoptosis in HeLa cells while suppressing oncogenic signaling pathways. Notably, this approach improved the efficacy of vincristine with low dose application (Asoudeh-Fard et al., 2025). Ab. RS22 suppresses HeLa cell proliferation by modulating PTEN/p53/Akt signaling pathways and activating caspase-3-mediated apoptosis (Asoudeh-Fard et al., 2024). Similarly, CH and KH inhibit HeLa cell growth by increasing BAX, caspase-8, and caspase-9 expression while reducing BCl-2, nuclear factor kappa B (NFkB) inhibitor, and RelA gene expression (Asoudeh-Fard et al., 2024). Lastly, *B. adolescentis* SPM1005-A demonstrates anti-HPV activity through suppression of E6/E7 oncogene expression (Cha et al., 2012).

#### 3.3.4 Prostate cancer

Prostate cancer (PCa) ranks among the most prevalent malignancies in men worldwide. Current non-surgical management strategies primarily include androgen deprivation therapy (ADT), radiotherapy (RT), ablation therapy, chemotherapy, and immunotherapy. However, these treatments often involve significant side effects and frequently encounter drug resistance in advanced disease (Evans, 2018). Consequently, developing novel adjuvant strategies to enhance efficacy and reduce toxicity has become a clinical research priority.

Probiotics demonstrate potential as adjuvant or alternative therapies for PCa through maintenance or restoration of healthy microbial communities, offering potential advantages of simplicity and cost-effectiveness. Research indicates probiotics directly interfer with biological behavior of prostate cancer cells. For example, A whey-based beverage containing specific probiotic strains-including *L. acidophilus* (La-05, La-03, and casei-01) and *Bifidobacterium* Bb-12-was demonstrated to inhibit the viability and induce apoptosis in PC-3 and DU-145 prostate cancer cells *in vitro*. Subsequent evaluation of the individual probiotic strains revealed that treatment with beverages fermented with Lc-01 or Bb-12 significantly increased apoptotic PC-3 cells compared to the control group. Additionally, the beverage containing La-05 also markedly reduced the viability of PC-3 cells and significantly enhanced their apoptotic rate. For DU-145 cells, these probiotic beverages similarly suppressed cell viability and elevated the rate of late apoptosis (Rosa et al., 2020). Another study investigated the effects of salicylic acid on the functional properties of *Lacticaseibacillus rhamnosus GG* (LGG) and evaluated the *in vitro* cytotoxicity of its combination with LGG against human colon and prostate cancer cells. The results demonstrated that salicylic acid significantly enhanced the co-aggregation capacity of LGG with Escherichia coli, as well as its antioxidant properties, while also inducing a cytotoxic effect of LGG against human colon cancer cells. These findings suggest that the interaction between LGG and salicylic acid may potentiate probiotic functionality (Celebioglu, 2021).

Probiotics can also modulate gut microbial composition in the host. Studies shown that probiotic supplementation promotes growth of beneficial bacterial communities, potentially reducing PCa risk in high-risk men (Fujita et al., 2022). As an androgen-dependent disease, PCa development is closely associated with androgen receptor activation, driving cell proliferation and survival, which makes inhibition of androgen synthesis a key therapeutic strategy (Nonnast et al., 2025). Research reveals that ADT depletes androgen-utilizing Corynebacterium spp., while oral abiraterone acetate administration further enriches health-associated AKK (Daisley et al., 2020). Furthermore, rectal volume has been identified as one of the most critical factors influencing prostate positioning during radiotherapy. Studies have shown that *Lactobacillus* supplementation not only effectively reduces the prostate volume control rate (PVCR) in prostate cancer radiotherapy but also mitigates intestinal gas production induced by chemotherapy. However, given that the long-term safety of its administration remains to be fully elucidated, excessive *Lactobacillus* supplementation may paradoxically precipitate adverse effects such as abdominal distension in patients (Ki et al., 2013). Inflammation also plays important roles in PCa pathogenesis, as exemplified by *L. reuteri* mitigating radiation-induced inflammation, potentially improving treatment outcomes (Nascimento et al., 2014).

### 3.4 Erectile dysfunction

Erectile dysfunction (ED) represents a prevalent health issue affecting approximately 12% of reproductive-aged couples globally, with male factors contributing to approximately 50% of all cases (Shamloul and Ghanem, 2013). This condition is associated with multiple risk factors including physical inactivity, smoking, alcohol or substance abuse, obesity, metabolic syndrome, and sleep disorders (Derby et al., 2000), clinically manifested as impaired sperm quality, sex hormone imbalances, and diminished sexual function. Recent research in microbiome have introduced the concept of the gut–testis axis, providing novel perspectives on the regulation of male reproductive health. Evidence indicates that probiotics participate in physiological functions of the male reproductive system through both direct and indirect mechanisms (Badran et al., 2023) (Table 3).

**Table 3:** Clinical trial evidence levels.

Disease	Random	Placebo	Group	Time	levels	References
PCOS	+	+	60	12 weeks	High-level evidence	Ostadmohammadi et al., 2019
	+	+	104	6 months	High-level evidence	Kaur et al., 2022
	+	+	60	12 weeks	High-level evidence	Kwok et al., 2022
BC	+	+	159	2.5 years	High-level evidence	Juan et al., 2022
	+	+	67	8 weeks	High-level evidence	Khazaei et al., 2023
Cervical cancer	+	+	89	12 weeks	High-level evidence	Xu et al., 2025a
	+	+	54	6 months	High-level evidence	Verhoeven et al., 2013
EMs	+	-	20	1 month	Moderate-level evidence	Bakun et al., 2023

Probiotics exert beneficial influences on ED by modulating hormonal levels and improving sperm function. Sex hormones play critical roles in male reproductive health. Testosterone, the primary androgen, not only participate in spermatogenesis and sexual function maintenance but also reflect fertility potential through alterations on sperm concentration, motility, and morphology. Research demonstrates that probiotics restore seminiferous tubule architecture, reverse arrested spermatogenesis, and ameliorate testicular dysfunction (Wu et al., 2024a). Additionally, probiotic supplement increases serum testosterone, FSH, and LH levels, enhances sperm kinematic parameters, and reduces the proportion of immotile sperm (Dardmeh et al., 2017). For instance, *B. longum* subsp. longum BL21 enhances reproductive capacity in zebrafish through hormonal regulation and sperm quality improvement (Dong et al., 2025b). Another subspecies B8762 upregulates reproduction-related genes including Etv4, Adamts16, Prok2, Gpr55, and Rad54b, restoring spermatogenic cell density and seminiferous tubule organization (Zhao et al., 2025). LGG ameliorates chronic unpredictable stress (CUS)-induced impairments in sperm count, motility, morphology, ultrastructure, DNA integrity, and chromatin condensation, while preventing CUS-induced testosterone alterations through upregulation of testicular StAR and P450scc expression (Guo et al., 2020). Further studies indicate that combined administration of *B. longum* with Cynara scolymus extract or *L. rhamnosus* CECT8361 yields superior outcomes in elevating LH and FSH levels, sperm concentration, and motility compared to individual treatments (Bozorgpoursavadjani et al., 2025; Valcarce et al., 2019).

Probiotics can also improve male reproductive function by suppressing oxidative stress, reducing inflammation, and modulating HPA axis. Oxidative stress and chronic inflammation represent key contributors to reproductive dysfunction. For example, polystyrene microplastics (PS-MP) induce HPG axis disruption, reduced reproductive hormone levels, testicular oxidative damage, and spermatogenic cell apoptosis due to excessive oxidative stress and p38 MAPK signaling activation, ultimately leading to infertility (Hwang et al., 2025; Xie et al., 2020). Studies demonstrate that probiotic supplement inhibits IL-17A signaling activation, attenuates inflammation, and ameliorates PS-MP-induced sperm quality deterioration (Zhang et al., 2023). Specifically, *L. brevis* GKJOY reduces oxidative stress and pro-inflammatory cytokine levels, restores hormonal balance. *L. brevis* GKJOY can also modulate neurotransmitter and effectively alleviates reproductive impairment in male rats (Hwang et al., 2025). LGG significantly enhances activities of catalase, glutathione peroxidase, and superoxide dismutase while reducing levels of oxidative products such as malondialdehyde and protein carbonyls, as well as downregulation of inflammatory mediators including cyclooxygenase-2, IL-1β, IL-6, and TNF-α, thereby blocking CUS-induced inflammatory and oxidative pathways (Guo et al., 2020). Furthermore, oral administration of *L. mesenteroides* SD23 improves obesity-associated metabolic dysfunction in high-fat diet-fed mice by upregulating TNF-α expression and modulating cholesterol, leptin, and glucose levels (Castro-Rodriguez et al., 2020).

The microbiome-gut-brain axis, a bidirectional communication system between the gastrointestinal tract and central nervous system, has been recently expanded to include testicular function, developing a new concept of the microbiome-gut-brain axis. Stress affects testicular function through activation of the HPA axis. For instance, restraint stress (RS) induces male reproductive defects via HPA axis activation and reactive oxygen species production (Akram et al., 2023). Research indicates that probiotics regulate HPA axis function in male animals and alleviate anxiety-like behaviors (Haas et al., 2020). *L. plantarum* improves hyperinsulinemia-induced reproductive dysfunction by modulating antioxidant status, lipid metabolism, and insulin signaling in the mouse HPA axis (Edem et al., 2021). Combination of fructo-oligosaccharides (FOS) with LGG NCDC-610 or *L. fermentum* NCDC-40 suppresses RS-induced HPA axis hyperactivation and enhances male fertility (Akram et al., 2023). Additionally, combined administration of *Levilactobacillus* 505 and Trifolium extract alleviates chronic mild stress induced testicular functional impairment through HPA axis modulation (Joung et al., 2022).

### 3.5 Clinical research of probiotics

PCOS represents a prominent area of current clinical research on probiotics. A number of randomized controlled trials and systematic reviews have demonstrated the benefits of probiotic supplementation in women with PCOS. Tabrizi et al. (2022) further suggested that probiotics may contribute to improvements in body weight, body mass index, and insulin levels, though no significant effects were observed on dehydroepiandrosterone sulfate, total cholesterol, low-density lipoprotein cholesterol, or high-density lipoprotein cholesterol. In a randomized, double-blind, placebo-controlled trial, Kaur et al. (2022) reported that multi-strain probiotic supplementation, when combined with dietary and lifestyle modifications, significantly promoted menstrual cycle regularity, reduced body weight, and improved metabolic and hormonal profiles in women with PCOS. Additionally, the impact of probiotics on inflammatory markers associated with PCOS has been investigated. In a 12-week intervention, 60 PCOS patients received daily supplementation with *L. acidophilus, L. plantarum, L. fermentum*, and *L. gasseri*. Results showed that probiotic supplementation significantly upregulated IL-10 expression and reduced IL-6 levels, compared to the placebo group, while no significant difference in TNF-α levels was observed between the groups (Kwok et al., 2022).

Probiotics have also demonstrated benefial effects on sex hormone-related malignancies. Juan et al. (2022) found that probiotic supplementation prevented chemotherapy-related cognitive impairment in BC patients by modulating plasma metabolites such as p-Mentha-1,8-dien-7-ol. Another study reported that an 8-week synbiotic intervention in 67 BC patients significantly reduced chemotherapy-associated complications, including bowel irregularities and fatigue, while symptoms such as nausea, vomiting, and anorexia were alleviated compared to baseline (Khazaei et al., 2023). Furthermore, probiotics have shown efficacy in ameliorating HPV-related symptoms. Study showed that 12 weeks of *L. plantarum* Probio87 supplementation significantly alleviated vulvar dryness, pain, and improved social interaction, daily activities, and sexual quality of life in HPV-positive women (Xu et al., 2025a). A six-month follow-up study further suggested that probiotics facilitated the clearance of cytological abnormalities in HPV-positive women with low-grade squamous intraepithelial lesions (Verhoeven et al., 2013).

The clinical application of probiotics has also been explored in other sex hormone-related disorders. One study investigated the adjunctive use of *Femina Probiz*, a probiotic product manufactured by Unic Biotech (India), in 20 patients with EMs. Following a one-month intervention, probiotics were found to induce multiple changes in endometrial lesions, most notably a significant upregulation of NLRP3 inflammasome mRNA expression (Bakun et al., 2023) (Table 4).

**Table 4:** The substrates and products of key enzymes.

Enzyme	Substrates	Products
GUS	Estrogen-glucuronic acid conjugate	Estrogen
3β-HSD	Testosterone/estrogen	Degraded/inactivated metabolites
5β-dihydroprogesterone reductase	Progesterone	Metabolites lacking progestogenic activity
HSDH	Free bile acids released after BSH conjugation	Secondary bile acids
Dehydroxylase	Primary bile acids	Secondary bile acids
SULT1E1	Estrogen	Sulfonated estrogen

Despite the promising clinical potential demonstrated by probiotics in conditions such as PCOS, sex hormone-related malignancies, and other gynecological disorders, the current clinical research exhibits limitations. Firstly, in the majority of clinical trials, probiotics have been administered primarily as an adjunctive strategy rather than as a standalone therapeutic intervention. Consequently, studies investigating the independent efficacy of probiotics remain scarce, rendering it difficult to delineate their direct effects. Secondly, while some studies have reported positive outcomes following probiotic administration, there has been insufficient attention paid to the documentation and systematic evaluation of adverse effects. A synthesis of available clinical data indicates that probiotics are generally safe in healthy populations. Nevertheless, a minority of recipients may experience adverse effects, including gastrointestinal discomfort such as diarrhea or constipation (Goldenberg et al., 2017), intestinal ischemia (Sotoudegan et al., 2019), and even endocarditis (Boumis et al., 2018). In high-risk groups including immunocompromised patients, individuals with severe intestinal disorders, those with compromised intestinal barrier function, and critically ill patients under intensive care, the use of probiotics warrants heightened vigilance due to the potential for complications (Neish, 2009). Furthermore, cautious evaluation is also required for infants with an underdeveloped intestinal barrier (Lin et al., 2023), as well as for pregnant women and cancer patients (Baldi et al., 2021). Given the vast diversity of probiotic strains and their complex mechanisms of action, clinical application should prioritize strains that are well-characterized, quality-controlled, and demonstrate a robust safety profile. As shown in Table 2, the daily intake of probiotics should reach 10^9^−10^11^ CFU, with an initial intervention period typically lasting 8 to 12 weeks. Regarding the safety of long-term use, further accumulation of follow-up data is needed. Additionally, when probiotics are co-administered with antibiotics, an interval of at least 2 h should be observed to prevent the inactivation of live probiotic organisms (Li et al., 2020). In summary, future investigations should not only explore the feasibility and efficacy of probiotics as standalone interventions but also design trials with safety as a primary endpoint, thereby enabling a more comprehensive assessment of their clinical value in this field.

### 3.6 The role of prebiotics, synbiotics, and fecal microbiota transplantation of sex hormone-related disorders

Prebiotics are a class of fermentable compounds primarily composed of unsaturated fatty acids, polyphenols, and carbohydrates (Singh et al., 2023). Unlike probiotics, prebiotics do not directly introduce live bacteria into the intestine. Instead, they exert indirect effects by promoting the growth of beneficial microbial populations, such as *Lactobacillus* and *Bifidobacterium* (Canfora et al., 2019). Evidence indicates that prebiotic intake significantly reduces serum levels of total cholesterol, triglycerides, LDL-C, glucose, hs-CRP, DHEA-S, and free testosterone in women with PCOS. Additionally, prebiotic supplementation elevates HDL-C levels and contributes to the regulation of menstrual cyclicity (Gholizadeh Shamasbi et al., 2019). Among patients with breast cancer, prebiotic administration improves select anthropometric parameters, although no significant effects are observed for others (Thu et al., 2023). Prebiotics modulate estrogen metabolism, immune function, and metabolic pathways, thereby offering potential avenues for breast cancer prevention and treatment (Sabit et al., 2025). A clinical study further revealed a negative association between dietary fiber intake and HPV infection (Zhang et al., 2021). Conversely, certain prebiotic sources—such as dairy products, dietary fats, and polyphenols—have been linked to a statistically significant increase in prostate cancer risk at specific concentrations (Mandair et al., 2014). Animal studies demonstrate that the prebiotic mannooligosaccharide influences the HPA axis, promotes seminiferous tubule maturation and spermatogenesis, and alters plasma corticosterone and testosterone levels, thereby affecting reproductive system development in mice (Poutahidis et al., 2014).

Synbiotics are combination formulations containing both probiotics and prebiotics, designed to exert synergistic effects (Senthilkumar and Arumugam, 2025). In women with PCOS, 12 weeks of synbiotic supplementation resulted in elevated levels of FAI, hs-CRP, and NO (Canfora et al., 2019). The concurrent administration of the prebiotic fructooligosaccharide and probiotics reduced anthropometric parameters, waist circumference, body fat percentage, and lymphedema volume in breast cancer patients (Thu et al., 2023). Moreover, combined intervention with gut microbiota and dietary fiber improved estrogen circulation and β-glucuronidase activity in postmenopausal women with breast cancer (Zengul et al., 2021).

Fecal microbiota transplantation (FMT) involves the transfer of fecal microbiota from a healthy donor into a patient's intestine, aiming to treat associated diseases through the reconstitution of the gut microbial community. In a rat model of PCOS, both *Lactobacillus* intervention and FMT ameliorated androgen levels and modulated insulin function (Guo et al., 2016). Using a mouse model of EMs, researchers found that FMT altered the composition of the gut microbiota in diseased animals (Ni et al., 2021). Furthermore, FMT enhanced the production of SCFAs, notably butyrate, and promoted T-cell expansion as well as the secretion of the anti-inflammatory cytokine IL-10. These changes help sustain intestinal immune homeostasis and facilitate recovery from cervical cancer (Tao et al., 2025). Dong and colleagues reported that ulcerative colitis leads to prostate enlargement and elevated GPER expression, changes that are reversed by FMT. Following FMT, butyrate levels in prostate tissue also increased. *In vitro* experiments further demonstrated that fecal material from healthy mice enhances GPER expression, inhibits cell proliferation, and induces apoptosis in prostatic hyperplastic cells (Dong et al., 2022).

In summary, interventions with microecological modulators represent a novel direction in the study of sex hormone-related diseases. Nevertheless, current evidence does not sufficiently support their adoption as a standard therapeutic regimen. Accordingly, further validation through high-quality randomized controlled trials is imperative.

## 4 Conclusion and outlook

This review systematically delineates the intricate and finely tuned bidirectional regulatory network known as the “gut microbiota-sex hormone axis.” This axis intricately links the gut microbiota with the endocrine system through multiple mechanistic layers, including the modulation of enzymatic activity, immune and inflammatory responses, metabolic processes, and the microbiome-gut-brain axis, collectively contributing to the maintenance of physiological homeostasis. The gut microbiota directly or indirectly metabolizes sex hormones, which in turn shape the composition and structure of the microbial community—a dynamic equilibrium essential for health.

Probiotics, as live microorganisms conferring health benefits, have demonstrated unique potential in modulating the gut microbiota-sex hormone axis. Preclinical and clinical studies have revealed that single or combined probiotic strains have been shown to improve conditions such as PCOS, EMs, hormone-related malignancies, and male reproductive dysfunction. However, current research has been limited by challenges too. First, the majority of trials are characterized by relatively small sample sizes, which constrains the generalizability of the findings. Second, probiotic intervention periods are typically confined to approximately 8 to 12 weeks, a duration that is insufficient to capture long-term effects, particularly given the temporal dynamics inherent in sex hormone regulation. The absence of extended follow-up data precludes a comprehensive assessment of both the durability and safety of probiotic efficacy.

Furthermore, probiotics exhibit strain-specific effects; however, studies frequently employ disparate strain combinations without one-to-one comparisons to identify optimal strains or synergies. This heterogeneity impedes direct comparison and synthesis of results across studies. Future research should prioritize high-quality randomized controlled trials with prolonged follow-up periods to elucidate underlying mechanisms and refine therapeutic strategies. With more comprehensive studies, probiotics could become a valuable component in the integrative management of sex hormone-related disorders, offering patients safer and more comprehensive treatment options.

## Glossary

### Abbreviations

3β-HSD, 3β-hydroxysteroid dehydrogenase; 5-HT, 5-hydroxytryptamine; GUS, β-glucuronidase; ACTH, Adrenocorticotropic hormone; AKK, *Akkermansia muciniphila;* ADT, Androgen deprivation therapy; AR, Androgen receptor; AhR, Aryl hydrocarbon receptor; ANS, Autonomic nervous system; *B. longum, Bifidobacterium longum;* BSEP, Bile salt export pump; BSH, Bile salt hydrolase; BC, Breast cancer; CDCA, Chenodeoxycholic acid; CCK, Cholecystokinin; CUS, Chronic unpredictable stress; CB, *Clostridium butyricum;* CRF/CRH, Corticotropin-releasing factor/hormone; CDK2, Cyclin-dependent kinase-2; DHT, Dihydrotestosterone; EC, Endometrial cancer; EMs, Endometriosis; ECs, Enterochromaffin cells; EECs, Enteroendocrine cells; ED, Erectile dysfunction; EcN, *Escherichia coli strain* Nissle; ER, Estrogen receptor; ERβ, Estrogen receptor β; ER^+^, Estrogen receptor-positive; FXR, Farnesoid X receptor; FMT, Fecal microbiota transplantation; FSH, Follicle-stimulating hormone; FOS, fructo-oligosaccharides; TGR5, Takeda G protein-coupled receptor 5; GPCRs, G protein-coupled receptors; GLP-1, Glucagon-like peptide-1; GDCA, glycine-conjugated deoxycholic acid; GnRH, Gonadotropin-releasing hormone; GnRHa, Gonadotropin-releasing hormone agonists; HDAC, Histone deacetylase; HPV, Human papillomavirus; HSDHs, Hydroxysteroid dehydrogenases; HPA, Hypothalamic–pituitary–adrenal; HPG, Hypothalamic–pituitary–gonadal; IFN-γ, Interferon-γ; IL-1, Interleukin-1; LGG, *Lacticaseibacillus rhamnosus GG; L. casei, Lactobacillus casei;* LH, Luteinizing hormone; MMP9, Matrix metalloproteinase-9; MGBA, Microbiome–Gut–Brain Axis; NK, Natural killer; NE, Nuclear factor kappa B; NFkB, Nuclear factor kappa B; PCOS, Polycystic ovary syndrome; PXR, Pregnane X receptor; PCa, Prostate cancer; PVCR, Prostate volume control rate; RT, Reactive oxygen species; ROS, Reactive oxygen species; RS, Restraint stress; pRb, Retinoblastoma protein; SeNP, Selenium nanoparticles; SCFAs, Short-chain fatty acids; SB, Sodium butyrate; SULT1E1, Sulfotransferase family 1E member 1; Th1, T helper type 1; TLRs, Toll-like receptors; TPH1, Tryptophan hydroxylase 1; TRAIL, Tumor necrosis factor-related apoptosis-inducing ligand; VN, Vagus nerve; VAS, Visual Analog Scale.
